# Physical activity and physical fitness in community patients with alcohol use disorders versus matched healthy controls: cross-sectional data from Uganda

**DOI:** 10.11604/pamj.2022.41.190.30673

**Published:** 2022-03-09

**Authors:** Davy Vancampfort, Samuel Kimbowa, Mats Hallgren, James Mugisha

**Affiliations:** 1 KU Leuven Department of Rehabilitation Sciences, Leuven, Belgium,; 2 Butabika National Referral and Mental Health Hospital, Kampala, Uganda,; 3 Department of Public Health Sciences, Karolinska Institutet, Stockholm, Sweden; 4 Faculty of Arts and Social Sciences, Kyambogo University, Kampala, Uganda

**Keywords:** Alcohol, physical activity, physical fitness

## Abstract

In order to develop adequate public health interventions, there is a need to explore whether people with an alcohol use disorder (AUD) not requiring inpatient treatment do have compromised physical health and are consequently a population at risk. We cross-sectionally compared physical fitness and physical activity levels in community patients with an AUD with healthy matched controls in Uganda. Fifty community patients (42 men, median age=32.0 years, interquartile range=10.7 years) and 50 age-, gender- and body mass index-matched controls performed a 6-minute walk test (6MWT), and completed the Simple Physical Activity Questionnaire (SIMPAQ). Differences between groups were assessed with a t-test or Mann Whitney U test when appropriate. Community patients with AUD have significantly lower 6MWT [median=480.0 (interquartile range=109) versus 802.5 (121.2) m, P<0.001], SIMPAQ walking [0 (30.0) min/day versus 35.0 (17.4) min/day, P<0.001], SIMPAQ exercise [0 (1.5) min/day versus 0 (2.5) min/day, P<0.001], and SIMPAQ incidental physical activity [30.0 (50.0) min/day versus 300.0 (315.0) min/day, P<0.001]. A reduced physical fitness and physical inactivity should be considered and assessed in early interventions targeting community patients with AUDs. If left untreated, both might also emerge as important modifiable risk factors for somatic co-morbidity in this population-at-risk.

## Introduction

Alcohol use disorders (AUD) are an important public health concern and globally a leading cause of disability accounting for almost 20% of all disability-associated burden, particularly in low-income countries where an adequate health care infrastructure is often lacking [1]. It is anticipated that the burden will increase even further in the decades to come, in particular, due to a high risk of co-morbid somatic disorders in this population, mainly in low-income countries [2]. Despite the recognition that AUD impose a tremendous public health burden in low-income countries, preventive and early interventions with a focus on mental and physical health needs are lacking in this part of the world [3]. Little to no attention is, for example, given to somatic health screening of people with AUD in Sub-Saharan Africa, or to public health interventions focusing on physical activity and physical fitness outcomes [4]. Consequently, there is a need to increase awareness that people with AUD in this part of the world should be considered a population-at-risk [3]. Two recent studies in inpatient care facilities from high-income countries [5,6] were the first to demonstrate that in patients with AUD have a lower physical fitness and are more physically inactive than the general population. However, data comparing physical fitness and physical activity levels in community patients with AUD not receiving inpatient treatment versus healthy matched controls are absent in the international literature and therefore recently calls were made to explore this in more detail in people with lower risk drinking and/or levels of alcohol dependence [5,6]. Moreover, data comparing physical fitness and physical activity levels of people with AUD versus the general population are completely absent in Sub-Saharan Africa. Exploring these differences in this part of the world is of relevance due to less awareness in these countries about the importance of physical activity and physical fitness levels as important health indicators, differences in access to primary health care with, among others a lack of access to appropriate care facilities, socio-cultural attitudes towards mental health with more stigma, and differences in environmental factors (e.g., safety and climate issues) making it more complicated to be physically active compared to high-income countries [4]. Therefore, we compared physical fitness and physical activity levels in community patients with AUD with healthy matched controls in Uganda.

## Methods

**Study design and setting:** this is a cross-sectional study in Butabika National Referral Mental Health Hospital in Kampala, Uganda.

**Study population:** over a 5-month period, all new adult patients with a DSM-5 diagnosis of AUD followed via a community outreach program were invited to participate. Controls were recruited via an advertising campaign in Butabika National Referral Mental Health Hospital and included personnel at all levels (maintenance personnel, administrative personnel, nurses) and their relatives or acquaintances in order to be able to include controls of all socioeconomic status levels. The age of the controls could be one year younger or older than a gender-matched patient while BMI could be one point lower or higher. Interested participants were screened for any significant cardiovascular, neuromuscular and endocrine disorders which might prevent safe participation in the study using the Physical Activity Readiness Questionnaire [7] and excluded if contra-indications were present.

**Data collection:** all participants performed a 6-minute walk test (6MWT) [8] to assess their physical fitness levels, and completed the Luganda version of the Simple Physical Activity Questionnaire [9]. The questionnaires were interviewer-administered.

**Statistical analysis:** continuous data were assessed for normality using the Shapiro-Wilk test. Since BMI was the only variable which was normally distributed, it was presented as mean ± standard deviation (SD). The other, non-normally distributed variables were presented as medians (interquartile range). Differences between groups were assessed with a Student-t or Mann Whitney U test when appropriate. A two-sided level of significance was set at P<0.05. The statistical package SPSS version 27.0 (SPSS Inc., Chicago, IL) was used to perform the analyses.

**Ethical considerations:** the study procedure was approved by the ethical committee of Mengo Hospital. All participants gave their informed written consent.

## Results

**Participants:** a total number of 57 community patients with AUD were invited. Six female patients declined to participate (i.e., were not interested). One patient was excluded due to a musculoskeletal disorder preventing safe participation. In total, 50 participants with AUD were included in the final analysis. These participants were compared with 50 age- and gender-matched controls (Table 1). None of the eligible controls had to be excluded.

**Table 1:** comparisons in 6MWT and SIMPAQ scores between Ugandan outpatients with alcohol use disorders and age- and gender-matched general population controls

Variables	Alcohol use disorder (n=50)	General population controls (n=50)	P*
Gender (M/F)	42/8	42/8	1.0
Age (years)	32.0 (10.7)	32.0 (12.2)	0.99
BMI (kg/m^2^)	23.1 (5.0)	22.5 (4.5)	0.17
6MWT score (m)	480.0 (109.5)	802.5 (121.2)	<0.001*
SIMPAQ sedentary (hours/day)	12.0 (4.0)	12.0 (3.0)	0.67
SIMPAQ walking (min/day)	0 (30.0)	35.0 (17.4)	<0.001*
SIMPAQ exercise (min/day)	0 (1.5)	0 (2.5)	<0.001*
SIMPAQ incidental PA (min/day)	30.0 (50.0)	300.0 (315.0)	<0.001*

* Significant when P<0.05. Data expressed as numbers (gender) or median (interquartile range). BMI = body mass index, 6MWT = 6 minute walk test, PA = physical activity, SIMPAQ = Simple Physical Activity Questionnaire

Differences in physical fitness and physical activity levels between patients and general population controls Our data (Table 1) show that community patients with AUD have significantly (all P<0.001) lower 6MWT, SIMPAQ walking, SIMPAQ incidental physical activity scores. There was no significant difference in time spent sedentary between both groups (P=0.67).

## Discussion

Our data are the first to demonstrate that in a Sub-Saharan African country, people with AUD with lower risk drinking and levels of alcohol dependence, i.e. community patients not requiring inpatient treatment, are significantly less physically fit and less physically active than the general population. These findings indicate that community programs for people with AUD should not only target alcohol abuse but also the physical health of this vulnerable population. Before clear recommendations can be formulated, future research should explore lifestyle interventions focusing on physical inactivity and lower physical fitness levels in people with AUD living within the community.

Some methodological limitations need however to be considered in order to interpret the data. First, the study included volunteers, and hence physical fitness and activity levels in our participants with AUD and in healthy controls may be an overestimation. Nevertheless, since there was a good response rate, a serious distortion of the results due to selection bias is rather unlikely. Another limitation was the use of a questionnaire to assess physical activity levels. Questionnaires to assess physical activity levels are prone to both systematic and random errors. Third, we were not able to explore the role of specific psychotropic medications, including antipsychotics and benzodiazepines in more detail. Fourth, our data are cross-sectional and cannot establish cause and effect.

## Conclusion

Our study is the first to establish that community patients with AUD are physically inactive and are less physically fit compared with healthy controls. Both aspects should be considered and assessed in early interventions targeting this population. If left untreated, both might also emerge as eminent modifiable risk factors for somatic co-morbidity in this vulnerable population.

### What is known about this topic

- Alcohol use disorders impose a tremendous public health burden in low-income countries;
- Little attention is given to somatic health screening of people with an alcohol use disorder in Sub-Saharan Africa.

### What this study adds

- People with an alcohol use disorder are significantly less physically fit and less physically active than the general population;
- Community programs for treating alcohol use disorder should not only target alcohol abuse but also the physical health consequences.
